# P178: The IPC program in West-Africa: how do you make it feasible and useful?

**DOI:** 10.1186/2047-2994-2-S1-P178

**Published:** 2013-06-20

**Authors:** B Ndoye

**Affiliations:** 1 Afrhyquasec, Dakar, Senegal

## Introduction

Africa generally, West-Africa in particular, is characterized by a remarkable lack of structured national IPC programs. There are numerous challenges to face, but there are also existing opportunities, which can enable to improve the current situation by using a rational and adapted approach.

## Objectives

The objective is to present the best way to implement IPC programs in Francophone West-Africa.

## Methods

The author propose a concrete way to implement increasingly a comprehensive national IPC program, based on main challenges (specially the lack of human resources), and the numerous opportunities as the many tools and guidelines currently proposed by WHO to face IPC, NGO’s supporting countries to strengthen the health system, and specially Senegalese experience.

## Results

Recommendations are based above all on two strategic issues:

- Human resources development

- Implementation of a national program, primarily based on basic processes (Standard Precautions).

## Conclusion

Starting national programs in West-Africa is feasible, useful and necessary, with currently available resources. That implies a political willingness of the countries and the support of development partners. These programs should firstly be based on basic processes of IPC, which are cross-cutting, involve all categories of healthcare workers, and don’t need specialized knowledge and skill. Starting from this entry point, the programs will be strengthened and adapted to local realities, in order to become increasingly really national programs for Patient Safety, with the human resources development.

All this will be achieved through a Senegalese project which aims at implementation of national patient safety programs in francophone West-Africa, starting with IPC activities.

## Disclosure of interest

None declared

